# Ultra-Processed Foods, Gut Microbiota, and Inflammatory Bowel Disease: A Critical Review of Emerging Evidence

**DOI:** 10.3390/nu17162677

**Published:** 2025-08-19

**Authors:** Amanda Luísa Spiller, Beatriz Gabriela da Costa, Ryan Nunes Yoshio Yoshihara, Enya Julia Zucari Nogueira, Natalia Salvador Castelhano, Andrey Santos, Maiara Brusco De Freitas, Daniéla Oliveira Magro, Ligia Yukie Sassaki

**Affiliations:** 1 Department of Internal Medicine, Medical School, São Paulo State University (Unesp), Botucatu 18618-686, SP, Brazil; a.spiller@unesp.br (A.L.S.); bg.costa@unesp.br (B.G.d.C.); ryan.nunes@unesp.br (R.N.Y.Y.); nutri.nacastelhano@gmail.com (N.S.C.); ligia.sassaki@unesp.br (L.Y.S.); 2 Institute of Biosciences, São Paulo State University (Unesp), Botucatu 18618-686, SP, Brazil; enya.julia@unesp.br; 3 Department of Internal Medicine, School of Medical Sciences, University of Campinas, Campinas 13083-887, SP, Brazil; andreysts@gmail.com; 4 Center for Molecular Prediction of Inflammatory Bowel Disease (PREDICT), Department of Clinical Medicine, Aalborg University (AAU), 2450 Copenhagen, Denmark; mbrusdefrei@dcm.aau.dk; 5 Department of Surgery, School of Medical Sciences, University of Campinas (UNICAMP), Campinas 13083-887, SP, Brazil

**Keywords:** ultra-processed food, gut microbiota, Crohn’s disease, ulcerative colitis, inflammatory bowel disease

## Abstract

Background/Aims: Inflammatory bowel diseases (IBDs), including Crohn’s disease (CD) and ulcerative colitis (UC), are chronic conditions marked by dysregulated inflammation in the gastrointestinal tract. Although the pathophysiology of IBD remains incompletely understood, it involves complex interactions between genetic predisposition and environmental triggers, such as gut microbiota imbalances and immune dysfunction, leading to chronic inflammation and mucosal injury. IBD affects approximately 7 million individuals globally, with prevalence increasing in Europe, North America, and Oceania. This rise parallels the growing consumption of ultra-processed foods (UPFs), which are typically rich in sugars, fats, and additives but low in fiber, vitamins, and other essential nutrients. These associations, this review critically examines the influence of UPF consumption on gut microbiota composition and function and its potential link to IBD. Methods: A bibliographic search was conducted in the SciELO, PubMed, and Cochrane databases. Results and Conclusions: High UPF consumption is associated with intestinal dysbiosis, marked by reduced microbial diversity, decreased short-chain fatty acid production, impaired barrier integrity, and mucus layer disruption. These alterations may promote immune-mediated diseases, including IBD, where dysbiosis is often characterized by an overgrowth of pathogenic bacteria such as *Clostridium* and *Enterococcus*, ultimately triggering inflammatory responses in the host.

## 1. Introduction

Over the years, various methods of obtaining, preserving, and preparing food have been developed to ensure survival in adverse environmental conditions [1]. The transition from nomadic societies to agricultural communities marked the beginning of a more direct relationship with food production, primarily based on minimally processed and seasonal ingredients. The Industrial Revolution brought about a profound transformation in food production, distribution, and consumption. The industrialization technologies enabled preservation, refining, large-scale production, and the creation of products with extended shelf life, enhanced convenience, and increased palatability. However, these advancements have shifted dietary patterns away from traditional diets and increased reliance on highly processed foods [1,2,3].

In recent decades, this phenomenon has intensified, particularly in developed countries and increasingly in developing nations, leading to the widespread adoption of Westernized dietary patterns [4]. These patterns are characterized by a high consumption of sugars, saturated fats, chemical additives, and ultra-processed foods (UPFs). Excessive UPF consumption may be intrinsically linked to low-grade inflammation. This is due to several factors, including large amounts of simple sugars, which can alter fructose metabolism, favoring insulin resistance and exacerbating low-grade inflammation [4,5]. Similarly, high concentrations of saturated and trans fatty acids can modify lipid structure, contributing to a proinflammatory environment. Furthermore, additives such as emulsifiers, colors, and artificial sweeteners can aggravate the inflammatory condition.

This significant dietary shift, coupled with an increasingly sedentary lifestyle, has been directly implicated in the escalating prevalence of non-communicable chronic diseases (NCDs), including type 2 diabetes, obesity, cardiovascular diseases, cancer, and various intestinal disorders [6,7,8,9]. Among these conditions, inflammatory bowel diseases (IBDs) are particularly notable, with incidence rising alarmingly even in regions historically characterized by low prevalence, such as Latin America, Asia, and Africa [10]. The etiopathogenesis of IBD is multifactorial, involving genetic predisposition, immunological alterations, environmental factors, and, critically, gut microbiota dysbiosis [11,12]. The observed rise in IBD incidence is occurring concurrently with the increased intake of UPF across various countries [13].

Considering this compelling evidence and the global impact of UPF consumption, this review aims to describe the role of UPF in the development of inflammatory bowel disease, explore its implications for gut microbiota composition and function, and discuss its potential associations with IBD risk.

## 2. Ultra-Processed Foods

**Figure 1:**
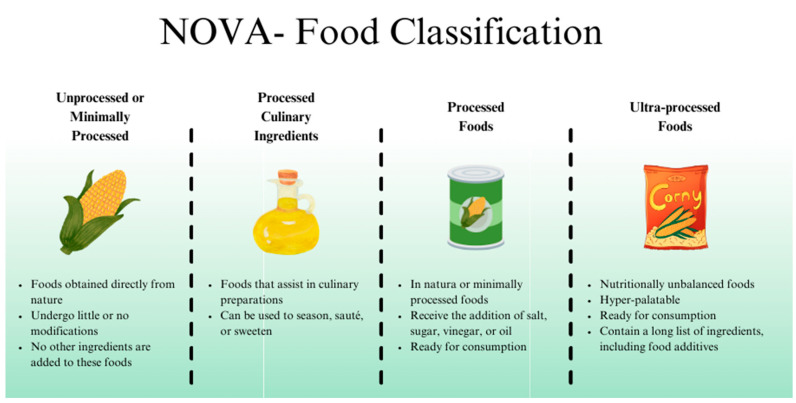
NOVA classification of foods [13].

According to the NOVA classification, foods are divided into four groups: natural or minimally processed, such as fruits and vegetables; processed culinary ingredients, such as oils, salt, and sugar; processed foods, which are products obtained from natural or minimally processed raw materials, with the addition of culinary ingredients, such as breads with natural yeast and 100% natural fruit juices; and ultra-processed foods, which are added with additives such as emulsifiers, sweeteners, preservatives, and colorings and may include components of natural foods, all characterized by a high degree of processing, such as sandwich cookies and frozen ready-to-eat meals [13,14] (Figure 1).

UPF accounts for up to 58% of daily caloric intake in high-income countries and approximately 30% in emerging economies. This widespread availability and consumption, particularly since the 1970s, coincides with a rise in NCDs, including IBD [15]. These products are highly palatable and nutritionally unbalanced due to their high content of refined sugars and fats (especially n-6 polyunsaturated fatty acids), low in fiber, vitamins, minerals, and other bioactive compounds, and identified as risk factors for the development of IBD [7,9]. Another significant concern is the use of food additives. Substances like stabilizers, colorants, and emulsifiers are added to alter foods’ physical, chemical, and sensory characteristics. Although approved by regulatory agencies, based on human studies, these compounds have been associated with alterations in the composition and function of the gut microbiota in animal studies [16,17,18,19].

While UPFs are associated with microbial imbalances and increased intestinal inflammation, diets rich in fiber and minimally processed foods promote greater microbial diversity, playing a role in preventing chronic inflammatory diseases [18]. However, fiber added to ultra-processed foods (UPFs) does not have the same physiological effects as fiber naturally present in whole foods, such as fruits and vegetables. In UPFs, fiber is an isolated ingredient, lacking the synergistic matrix of vitamins, minerals, and phytochemicals. The overall composition of UPFs—high in sugars, fats, and sodium—can neutralize any potential benefits of added fiber. Furthermore, fiber enrichment is often used as a marketing tool (health washing) to mask poor nutritional quality. For example, sugary breakfast cereals may be promoted as “high in fiber” despite being nutritionally inadequate. Critically, the gut microbiome of individuals consuming diets rich in UPFs may lack key microbes that degrade fiber, limiting the physiological benefits of added fiber. Studies on the ancestral microbiota suggest that UPF consumption impairs gut health, regardless of fiber content [18,20]. Thus, the increasing consumption of UPFs, paralleling the rising incidence of IBD in many countries, raises serious concerns about their impacts on gut health and systemic inflammation [12].

## 3. Impact of Ultra-Processed Foods on Gut Microbiota and Intestinal Homeostasis

The intestinal epithelial barrier is composed of a complex multifunctional structure that includes epithelial cells, intercellular junctions (such as tight junctions, and desmosomes), mucus, antimicrobial peptides, and the mucosa-associated immune system. Epithelial cells form a single layer that lines the intestinal lumen and act as a selective interface between the external environment and the internal milieu. Tight junctions regulate paracellular permeability, preventing the passage of microorganisms and toxins while allowing the absorption of nutrients and ions. Furthermore, the mucus secreted by goblet cells forms a physical barrier that hinders the adhesion of pathogens, and antimicrobial peptides, such as defensins, contribute to local immune defense [21,22].

The gut microbiota plays a vital role in human health, constantly adapting to its intestinal environment. It provides numerous physiological benefits in a “healthy state”, known as eubiosis or normobiosis. These processes include metabolizing dietary nutrients, degrading and fermenting fibers, synthesizing vitamins B12, B6, folate, and K, secreting antimicrobial compounds, modulating immune responses, and maintaining the integrity of the intestinal epithelial barrier to prevent pathogen adhesion and colonization [23,24]. However, the consumption of UPF is consistently linked to significant alterations in gut microbiome composition and low-grade inflammation [23,24]. Specifically, an increase in microbial taxa associated with obesity and metabolic disorders is observed, alongside a decline in beneficial groups like *Bacteroides*, *Verrucomicrobia*, *Eubacterium rectale*, *Coprococcus coccoides*, and *Bifidobacterium* [15]. This dysbiotic shift often produces a proportional rise in proinflammatory phyla such as Bacillota and Pseudomonadota. This imbalance in altered microbiota composition, combined with other triggers such as increased exposure to air pollution and higher levels of stress, for example, contributes to increased levels of proinflammatory cytokines, including interleukin-1 (IL-1), interleukin-6 (IL-6), and tumor necrosis factor (TNF-α), and lipopolysaccharides (LPSs) drive hyperinsulinemia, adipogenesis, and hepatic steatosis [25,26].

The persistent low-grade inflammation driven by UPF consumption can also lead to a “leaky gut” or increased intestinal permeability [5,18]. This compromised barrier then facilitates the translocation of pathogenic bacteria and LPS into the bloodstream, significantly exacerbating the inflammatory response observed in IBD. The hypothesis is that consuming more than 20% of calories or more than five servings per day causes changes in the gut microbiota. Furthermore, consuming 10% of calories from ultra-processed foods per day is associated with a 19% increased risk of developing Crohn’s disease [7,18]. LPS plays a central role by activating the innate immune system, triggering the recruitment of macrophages, neutrophils, and dendritic cells through Toll-like receptor (TLR) signaling, which in turn releases key proinflammatory mediators, such as IL-1α, IL-1β, TNF-α, and IL-6 [7,27,28]. For instance, serum LPS levels are reportedly six times higher in patients with active [7,26,27] Crohn’s disease (CD) compared to healthy controls, highlighting its potential to intensify CD-related inflammation [27]. Some studies suggest that the IL-23/IL-17 axis, central to CD pathogenesis, may increase the intestinal epithelium’s susceptibility to LPS exposure, further exacerbating inflammation [29]. While the evidence directly linking UPF to UC is less consistent—possibly due to its complex pathogenesis—circulating LPS may still act as a precursor in UC progression, as highlighted in a comprehensive review primarily encompassing experimental and observational studies that discussed this association [29].

Beyond LPS, flagellin levels have also been associated with CD inflammation and intestinal barrier integrity, as demonstrated in a comparative observational case–control study including 497 individuals with IBD (256 with Crohn’s disease, 207 with ulcerative colitis, and 34 with unclassified IBD) from the 1000 IBD cohort in Groningen, the Netherlands, using high-throughput phage immunoprecipitation sequencing (PhIP-Seq) [30]. Although its exact mechanism is not fully elucidated, the hypothesis linking flagellin to CD pathogenesis suggests it may drive T-cell and B-cell immune responses, potentially arising from compromised transepithelial electrical resistance to proinflammatory cytokines after flagellin exposure, leading to reduced tight junction proteins [31].

Emerging evidence suggests that habitual consumption of ultra-processed foods (UPFs) may aggravate the progression of intestinal inflammation and contribute to colon carcinogenesis through multiple interconnected mechanisms. UPFs, rich in food additives, emulsifiers, saturated fats, organic sugars, and specifically proinflammatory compounds, have been associated with intestinal dysbiosis, with reduced microbial diversity and increased pathogenic microorganisms. This alteration in the microbiome contributes to intestinal epithelial barrier dysfunction, facilitating endotoxin translocation and activating chronic inflammatory pathways, such as NF-κB signaling. These factors create a microenvironment conducive to genomic instability, uncontrolled cellular proliferation, and the evasion of apoptosis mechanisms, favoring the progression of chronic inflammation to dysplasia and carcinoma [17,32,33].

## 4. Impact of Food Additives on Gut Microbiota

Food additives, commonly found in UPF, are designed to enhance product characteristics and extend shelf life. However, a growing body of evidence indicates they can profoundly impact gut microbiota composition and function, thereby contributing to intestinal inflammation and dysbiosis. Studies in both humans and animals show that these additives can trigger proinflammatory changes in the microbiota, enhance LPS release, disrupt the production of beneficial metabolites like short-chain fatty acids (SCFAs), and impair the mucus layer and β-defensin expression [16,17,33]. Indeed, diets in which UPF constitutes over 20% of total daily caloric intake may compromise mucus barrier integrity, impair goblet cell function, and increase the risk of disease [34,35] (Table 1).

### 4.1. Emulsifiers

Emulsifiers are additives widely used in ultra-processed foods to improve texture, stability, and palatability. However, recent studies have raised concerns about the adverse effects of emulsifiers on gut health and their potential impact on the gut microbiome [40]. In vitro study utilizing fecal samples from healthy individuals has investigated the effects of common emulsifier dietary on the gut microbiome, including propylene glycol monostearate (PGMS) and sodium stearoyl lactylate (SSL). These latter two, frequently found in kinds of margarine, baked goods, and desserts, significantly reduced butyrate-producing families such as *Clostridiaceae*, *Lachnospiraceae*, and *Ruminococcaceae*, while increasing *Bacteroidaceae* and *Enterobacteriaceae*. SSL was also linked to elevated LPS and flagellin levels, possibly due to increased production by Escherichia [44].

Clinical studies in humans demonstrate that consuming 15 g per day of carboxymethyl cellulose (CMC) by healthy individuals induces significant changes in microbial composition [40]. Specifically, carboxymethylcellulose (CMC) may promote bacterial adherence to the intestinal epithelium, facilitating bacterial proliferation and infiltration between intestinal villi. It can also alter the gut microbial composition, decreasing beneficial species such as *Faecalibacterium prausnitzii* and *Ruminococcus* spp. (both associated with SCFA production), increasing *Roseburia* spp. and *Lachnospiraceae bacterium* [40,41]. Furthermore, consuming CMC and the emulsifier polysorbate 80 (P80) has been associated with elevated levels of Pseudomonadota, which promote mucosal inflammation. These changes can also increase bacterial adherence, penetration into the mucus layer, and migration toward intestinal crypts, amplifying the proinflammatory potential and promoting chronic inflammation [45]. P80 intake may even facilitate the translocation of bacteria, such as Escherichia coli, through M cells and Peyer’s patches, thereby compromising intestinal barrier integrity [46,47].

Xanthan gum (INS 415), an extracellular polysaccharide from Xanthomonas campestris, is a common food additive. Recent studies suggest that human gut microbiota can partially degrade xanthan gum [38,40]. Consumption of 214 mg/kg/day in adults, although safe, can cause symptoms of flatulence and abdominal pain [48]. Individuals with Westernized gut microbiota often harbor specific bacterial species, particularly from the Ruminococcaceae family, that can metabolize xanthan gum into oligosaccharides, which are subsequently utilized by *Bacteroides intestinalis*, forming a microbial food chain. This degradation can lead to the production of SCFAs, including butyrate, which is known for its anti-inflammatory and gut-protective actions [49]. However, the effects are not uniformly beneficial. Other studies suggest that xanthan gum can induce alterations in the microbiota, promoting the proliferation of opportunistic bacteria, such as *Ruminococcus gnavus* and members of the Enterobacteriaceae family, both of which are associated with intestinal inflammation [49]. For instance, a study on Wistar rats administered xanthan gum found a significant increase in inflammatory cytokines (IL-1β and TNF-α) and harmful modulation of epithelial tight junction proteins, such as Claudin-2, suggesting that at high doses or in predisposed individuals, xanthan gum may compromise intestinal barrier integrity and foster an inflammatory environment [39,49].

### 4.2. Non-Caloric Artificial Sweeteners

Non-caloric artificial sweeteners (NASs) are widely used for their calorie-free sweetness and have been implicated in adverse gut health outcomes. Suez et al. [36] observed that NAS intake impairs glucose tolerance in both humans and rodents, and results suggest that NAS-induced glucose intolerance is mediated through alterations to the commensal microbiota, with contributions from diverse bacterial taxa, and with changes directly linked to the gut microbiota, including an overgrowth of *Bacteroides* and a reduction in Bacteroidota, particularly Clostridiales species. The artificial sweeteners involved in changing glucose tolerance are saccharin, sucralose, and aspartame, with more pronounced effects from saccharin, including alterations in gut microbiota and an increased inflammatory potential, suggesting intestinal barrier dysfunction and elevated inflammatory markers such as TNF-α and IL-6 [3,36].

### 4.3. Maltodextrin

Maltodextrin, a widely used polysaccharide additive, improves food texture, flavor, and stability. However, frequent consumption may be linked to changes in microbial phenotype and host antibacterial defenses [50]. A study by Laudisi et al. [37] observed that rats given a 5% maltodextrin solution in water for 45 days exhibited a reduction in intestinal mucin 2 (Muc-2) and an increase in epithelial adhesion of pathogenic bacteria, which could contribute to susceptibility to intestinal diseases [37].

A systematic review of randomized, placebo-controlled human trials on maltodextrin found that nine studies (n = 10/21, 47.6%) reported induced changes in Firmicutes. Of these, six studies observed a significant increase in several members of the Firmicutes group after MDX doses ranging from 0.5 to 15 g/day for 1 to 16 weeks. However, none of the analyzed studies reported the Firmicutes: Bacteroidota ratio, which is a marker of gut dysbiosis [51].

### 4.4. Carrageenan

Carrageenan (CGN) is a natural polysaccharide extracted from red algae, widely used in the industry for its gelling, thickening, and stabilizing properties [52]. However, its use has been questioned due to its potential breakdown in the gastrointestinal tract, leading to the formation of degraded carrageenans (dCGNs), which may pose health risks, such as small bowel tumors, large bowel ulcers, and glucose intolerance [53]. An in vitro study by Han et al. observed that the formation of dCGNs by the intestinal microbiota significantly increased the production of nitric oxide (NO2), cyclooxygenase-2 (COX-2), and proinflammatory cytokines like IL-1β, TNF-α, and IL-6, which suggests a possible link to the development of IBD [43].

### 4.5. Synthetic Colorants (Azo Dyes)

Synthetic colorants have experienced a dramatic 500% increase in use over the past five decades, primarily to enhance product appearance [54,55]. These colorants, typically derived from petroleum, release reactive or toxic aromatic amines when metabolized in the gut. These metabolites possess carcinogenic potential and are associated with other chronic diseases [55,56,57]. Azo dyes (AZo), including Allura Red, Sunset Yellow, Amaranth, Tartrazine, Red 40, and Yellow 6, though permitted in food, have been linked to microbiota-related disease pathophysiology. The study by He et al. [35] was an experimental follow-up study in an animal model, in which the authors conducted an elegant series of in vitro and in vivo experiments to establish the necessity of bacterial processing and to identify the active metabolite. Consistent findings that both Red 40 and Yellow 6 induce colitis, and that inactivation of the azo bonds abolishes their colitogenicity, reinforce the credibility of the proposed mechanism [57]. The Lachnospiraceae family, including species like *Clostridium symbiosum* and *Hungatella effluvi*, appears to be a primary group capable of reducing AZo [56]. He et al. administered Red 40 and Yellow 6 to genetically susceptible rodents. They found that commensal bacteria like Bacteroides ovatus and Enterococcus faecalis metabolized these dyes to form sodium salt 1-amino-2-naphthol-6-sulfonate (ANSA-Na) [35]. This metabolite was then implicated in colitis induction by activating pathogenic CD4+ T cells through the IL-23 axis, stimulating IFN-γ production, and intensifying intestinal inflammation in response to exposure to Red 40 and Yellow 6 [35].

### 4.6. Nanoparticles and Microparticles

Nanoparticles are increasingly used to enhance product characteristics such as color, texture, and appearance while improving stability and shelf life [17]. An in vitro study evaluating the absorption and biological impact of iron oxide (E172) nanoparticle subtypes (yellow, orange, red, and black) found that all variants interacted with intestinal barrier cells. However, none demonstrated cytotoxic effects at concentrations below 100 μg Fe/mL after 24 h of exposure [42].

Commonly used microparticles include titanium dioxide (TiO_2_-E171), a whitening agent; aluminum silicate (AlSi-E559), an anti-caking agent; and silicon dioxide (E551) [17]. In 2023, the European Food Safety Authority raised concerns about the genotoxic potential of TiO_2_. Despite this, its use remains “authorized pending further review” by the Joint FAO/WHO Expert Committee on Food Additives (JECFA) [58]. TiO_2_ can alter the intestinal environment by reducing the expression of Muc-2, a key protein in mucus layer formation, directly affecting goblet cell function, impairing mucus production, and compromising intestinal barrier integrity [38]. In vitro research indicates that goblet cells efficiently absorb TiO_2_, which may interfere with their ability to secrete mucus effectively [38]. Rodent studies show that TiO_2_ ingestion leads to its uptake by intestinal cells, initiating a proinflammatory cascade. Prolonged exposure has been linked to the release of reactive oxygen species (ROS), resulting in altered gene transcription and promoting dysplasia [17,59,60].

In this context, the harmful effects of UPF components, such as emulsifiers, sweeteners, synthetic colorants, and nanoparticles, are evident through their ability to induce inflammation, alter the gut microbiome, and reduce populations of beneficial microorganisms, thereby possibly contributing to the development of IBD.

## 5. Dysbiosis and Modulation of the Intestinal Microbiota in IBD

### 5.1. Microbial Dysbiosis in IBD

**Figure 2:**
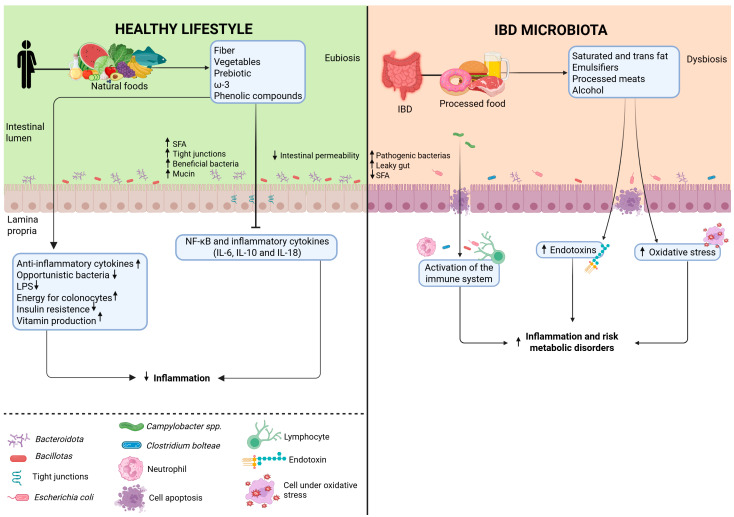
Green represents microbiota in eubiosis resulting from a healthy lifestyle. Red represents microbiota in dysbiosis due to a Western diet high in ultra-processed foods. The consumption of whole and/or minimally processed foods is associated with the promotion of an eubiotic environment characterized by increased production of short-chain fatty acids (SCFAs) and mucin, reduced intestinal permeability, lower levels of opportunistic bacteria and inflammatory factors, and elevated anti-inflammatory markers. In contrast, consuming ultra-processed foods contributes to dysbiosis, characterized by an increase in pathogenic bacteria, proinflammatory mediators, and metabolic disturbances. LPS: Lipopolysaccharide; NF-kB: Nuclear Factor Kappa B; IL-6: Interleukin-6; IL-10: Interleukin-10; IL-18: Interleukin-18; SFA: Saturated fatty acid; ω-3: omega-3 fatty acids. All black arrows indicate an increase or decrease in the product.

IBD is strongly linked to gut dysbiosis, a microbial imbalance characterized by an overgrowth of pathogenic bacteria such as *Clostridium* and *Enterococcus* [32]. This microbial alteration compromises mucosal integrity, increases intestinal permeability, and facilitates bacterial translocation, ultimately leading to the intense activation of host inflammatory responses [32]. In individuals with IBD, a significant reduction in the phyla Bacillota (formerly *Firmicutes*) and Bacteroidota (formerly *Bacteroidetes*) is commonly observed [61]. This decrease impairs the modulation of inflammatory responses and the production of SCFAs. Conversely, opportunistic pathogens, including certain Pseudomonadota (formerly Proteobacteria) species, proliferate and can exacerbate underlying inflammatory conditions (Figure 2) [62,63]. Studies, such as that by Santana et al. [64], confirm that individuals with IBD show a decreased abundance of beneficial bacteria and an increased presence of species that contribute to ongoing mucosal damage (Table 2 and Table 3).

SCFAs, particularly acetate, propionate, and butyrate, are microbial fermentation products with significant therapeutic relevance for human health. However, their levels are often altered in individuals with IBD [65]. Among these, butyrate is one of the most extensively studied in this population. It is produced by bacteria such as *Roseburia* spp., *Eubacterium rectale*, *Faecalibacterium prausnitzii*, and *Clostridium leptum* (phylum Bacillota), as well as *Akkermansia muciniphila* (phylum Verrucomicrobia). Butyrate is the primary energy source for intestinal epithelial cells and supports cell signaling, proliferation, and differentiation. In addition, it has strong anti-inflammatory effects mediated through the activation of G-protein-coupled receptors [18,20,66,67].

SCFAs also play a critical role in modulating immune responses by inhibiting nuclear factor kappa B (NF-κB) and histone deacetylases, thereby regulating innate and adaptive immunity [68]. These mechanisms support the restoration of tissue damaged by inflammation [65]. In patients with ulcerative colitis treated with infliximab, enzymes involved in butyrate oxidation (excluding SLC16A1) were found to be elevated in those who responded to biological therapy. These findings suggest that mucosal inflammation interferes with butyrate oxidation [69]. Consequently, the results indicate a correlation between inflammation and gene suppression in SCFA absorption and metabolism.

Obesity and diets high in trans and saturated fats, emulsifiers, processed foods, and alcohol contribute to elevated endotoxin levels, oxidative stress, and the expansion of pathogenic bacterial populations while reducing SCFA production. These changes promote inflammation and increase the risk of other comorbidities, including diabetes, metabolic-dysfunction-associated steatohepatitis (MASLD) [70], and various cardiovascular and neurological conditions [71,72,73].

### 5.2. Gut Microbiota Modulation in IBD

Modulating the gut microbiota has emerged as a key strategy in managing IBD^9^ and other chronic conditions [74,75,76]. Various factors, including long-term dietary patterns, physical activity, smoking, sleep quality, alcohol consumption, stress, medication use, and aging, influence the gut microbiota composition [77]. Several approaches are employed to promote intestinal microbiota modulation in individuals with IBD, described below. However, despite promising results in vitro and in experimental models, robust clinical evidence supporting their effectiveness in IBD patients remains limited.

### 5.3. Dietary Patterns

The Mediterranean diet is a well-researched dietary pattern for its health-promoting effects [78,79]. Rich in fiber, vitamins, minerals, and bioactive compounds, it is associated with a lower risk of IBD development and improved inflammatory markers, alongside a protective effect on the intestinal mucosa [80,81]. Its benefits include reducing proinflammatory bacterial species (*Escherichia coli*, *Ruminococcus gnavus*), increased production of SCFAs, fecal secretory immunoglobulin A (sIgA), and enhanced microbiota diversity and intestinal barrier function [82,83,84]. Clinical studies support its efficacy. Dogan et al. [85] observed reduced inflammatory biomarkers in UC patients, and Chicco et al. [86] noted clinical and quality-of-life improvement. Conversely, high consumption of UPF is associated with impaired metabolic and intestinal health, leading to increased inflammation and dysbiosis [87].

### 5.4. Prebiotics

Prebiotics are substrates that are selectively utilized by host microorganisms, providing a health benefit [88]. Examples of such substrates include pectin, inulin, and resistant starch, which can be naturally present in foods such as fruits [89,90]. While the European Society for Clinical Nutrition and Metabolism (ESPEN) suggests potential as an adjunctive therapy in IBD, evidence remains limited [91]. A Cochrane systematic review found insufficient evidence to support their efficacy for induction and maintenance of remission in mild-to-moderate UC, likely due to few studies [92].

### 5.5. Probiotics

Probiotics are live microorganisms that confer health benefits to the host when administered adequately [88,93]. A 2020 Cochrane review analyzing probiotics for maintaining remission in UC found no significant differences compared to other interventions [94]. ESPEN advises against their use for inducing or maintaining remission in CD, as they are generally ineffective [91]. However, probiotics may be considered for mild-to-moderately active UC and are recommended to prevent pouchitis. Despite these indications, current evidence does not support their routine use in clinical practice [95].

### 5.6. Symbiotics

A symbiotic is a mixture containing live microorganisms and substrates selectively utilized by host microorganisms, offering a health benefit [96]. A meta-analysis suggests that symbiotics may serve as an adjunctive therapy for moderate UC [97], but the limited number of studies necessitates further research. Preclinical studies show promise, but human studies have small sample sizes [98,99].

### 5.7. Postbiotics

Postbiotics are preparations of inactivated microorganisms and/or their components that confer a health benefit to the host [100]. Studies suggest postbiotics may aid immune modulation via SCFA production, improve intestinal barrier integrity, promote intestinal homeostasis, and inactivate inflammatory pathways [101]. However, most studies with IBD are preclinical or in vitro, with heterogeneous outcomes, underscoring the need for more robust clinical trials [102].

### 5.8. Fecal Microbiota Transplantation (FMT)

Fecal microbiota transplantation (FMT) involves transferring stool from a healthy donor to a recipient’s intestinal microbiota [103]. Primarily studied in recurrent *Clostridium difficile* infections and selected metabolic disorders [102,104,105,106], a Cochrane review suggests FMT may be a promising therapeutic option for inducing remission in mild-to-moderate active UC [107]. Nevertheless, further clinical trials are essential before FMT can be routinely recommended for treating IBD [95].

## 6. The Complex Relationship: UPF, Gut Microbiota, and IBD

The interplay between diet, gut microbiota, and IBD has been a significant research focus in recent decades [108]. Among various dietary factors, UPF has emerged as a critical contributor to gut dysbiosis and the exacerbation of inflammatory conditions, including CD and UC [108].

Chronic and high intake of UPF is consistently associated with a proinflammatory and pro-oxidant metabolic profile [26] mediated through interconnected physiological pathways, including elevated ghrelin levels, reduced peptide YY (PYY) levels, endothelial dysfunction, altered glucose metabolism, hormonal imbalances, and insulin resistance [109]. Furthermore, a higher prevalence of procarcinogenic bacteria is observed in the gut microbiota, whose metabolites can induce genotoxic DNA damage [18]. These microbial alterations may also compromise the gut–brain axis, potentially impairing cognitive function and mental health [18,110]. Elevated UPF consumption, especially in older adults, has been associated with reduced antioxidant enzyme activity (e.g., catalase and superoxide dismutase) and increased xanthine oxidase levels [111]. These findings indicate increased oxidative stress and diminished antioxidant defenses, accompanied by higher levels of proinflammatory cytokines, such as TNF-α, IL-6, and IL-15, and increased production of reactive oxygen species (ROS) [110]. The heightened activity of myeloperoxidase (MPO), a pro-oxidant enzyme, further contributes to this pro-oxidative state [111]. These mechanisms may exacerbate endothelial dysfunction, thereby increasing the risk of intestinal ischemia, particularly in vulnerable populations [109,111]. Supporting these observations, a positive association between UPF consumption and IL-6 levels has been found in cohorts from diverse socioeconomic backgrounds [39]. Ultimately, frequent UPF intake is implicated in IBD development and progression by promoting excessive ROS production and perpetuating oxidative stress in intestinal tissues. This oxidative environment directly contributes to cellular dysfunction and can induce necroptosis, thereby amplifying the inflammatory response and exacerbating the clinical course of IBD [112,113].

Excessive UPF intake is linked to reduced beneficial bacteria, such as *Faecalibacterium prausnitzii*, and decreased production of butyrate, an SCFA essential for maintaining intestinal barrier integrity, as discussed previously [40,45]. A healthy gut microbiota is crucial for immune modulation and intestinal homeostasis, thereby preventing chronic inflammation and protecting against infections. In IBD, this homeostasis is frequently disrupted, leading to dysbiosis marked by reductions in beneficial commensals (e.g., Bacillota and Bacteroidota) and increases in intestinal pathogens (e.g., *Escherichia coli* and Pseudomonadota) [114]. A disrupted microbiota in IBD is associated with elevated proinflammatory cytokines (including TNF-α and IL-6) and the activation of inflammatory signaling pathways like nuclear factor kappa B (NF-κB), contributing to sustained gastrointestinal inflammation [114].

**Figure 3:**
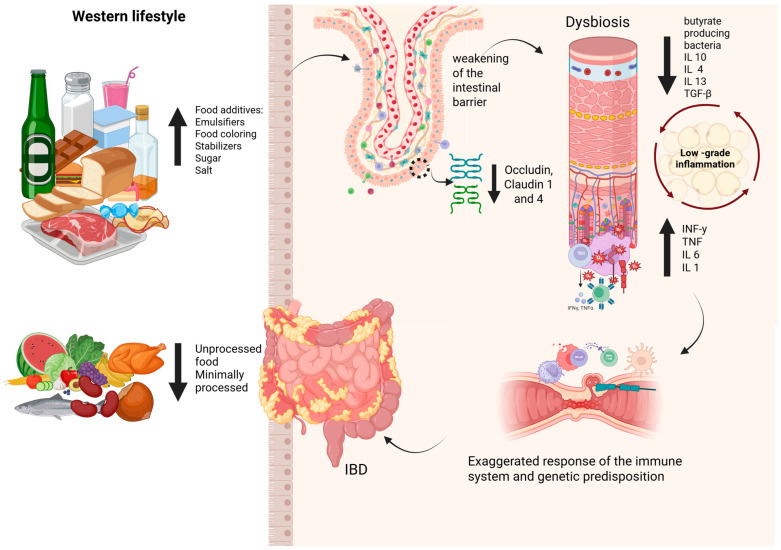
The Western diet’s impact on promoting dysbiosis, increasing intestinal permeability, and initiating the inflammatory cascade is a contributing risk factor for developing inflammatory bowel diseases (IBDs). IL 10: Interleukin-10; IL 4: Interleukin-4; IL 13: Interleukin-13; TGF-β: Transforming Growth Factor beta; IL 6: Interleukin-6; IL 1: Interleukin-1; IFN-γ: interferon gamma; TNF-α: Tumor Necrosis Factor alpha.

External influences, particularly diets high in UPF, exacerbate the proinflammatory state, which favors pathogenic bacteria growth and promotes excessive cytokine production [25]. The chronic inflammatory state stemming from UPF intake appears to be a key pathophysiological mechanism contributing to IBD development, particularly in CD. This disruption facilitates bacterial translocation and microbial imbalance, which activate an exaggerated immune response when combined with genetic predisposition, thereby increasing the risk of IBD onset [17,115,116,117] (Figure 3).

A robust cohort study involving 245,112 participants in the United States identified 857 IBD cases [104]. High UPF intake was associated with an increased risk of developing CD, linked explicitly to ultra-processed bread, breakfast cereals, and ready-to-heat frozen meals [104]. This association may be attributed to the high levels of carrageenan, synthetic emulsifiers, and sodium chloride in these products, which disrupt the intestinal barrier by promoting the proliferation of proinflammatory bacteria within the Pseudomonadota phylum [104]. While this process impairs epithelial integrity and reduces the mucus layer, favoring colitis development, the study found no statistically significant evidence for an association between UPF consumption and UC onset [104].

Conversely, a 2021 study tracking over 150,000 participants across 31 countries found that higher UPF intake was associated with an increased risk of both CD and UC. However, gut microbiota composition was not assessed [117]. Consumption of UPF, particularly processed meats and soft drinks, was more prevalent in North America, Europe, and South America [117]. However, no food group or additive was independently linked to a higher risk of IBD, emphasizing that overall dietary patterns significantly influence disease pathogenesis [117]. Interestingly, a study in a Caucasian population from eight European countries (over 400 participants, 13.2-year follow-up) found no association between increased UPF consumption and a greater IBD risk [118]. Instead, whole and minimally processed foods appeared to protect against CD, with no significant association for UC incidence [119].

Beyond incidence rates, research is increasingly focusing on the role of diet in disease progression [104]. Preda et al. [119] conducted a clinical trial of 168 participants with CD/UC. It showed sustained remission in 95.2% of a low-UPF intervention group versus 85.7% in a control group (*p* = 0.036), after one year, underscoring the role of lifestyle habits in IBD management [119]. Observational studies also suggest an inverse association between high fiber intake and CD risk, though UC results remain inconclusive [119]. Evidence suggests that habitual consumption of UPFs contributes to intestinal dysbiosis and inflammation, potentially influencing disease onset and progression. However, during remission, diverse dietary patterns rich in whole foods and fiber promote eubiosis and intestinal barrier maintenance. Conversely, excessive consumption of insoluble fiber can aggravate symptoms and compromise nutritional status, requiring specific dietary adaptations during disease activity. This duality illustrates the complexity and paradoxical nature of the relationship between diet and IBD [7,9]. The maintenance phase of the diet is crucial for developing and maintaining healthy eating habits, reinforcing the importance of diet for gut health and microbiome. Several dietary strategies, such as the CD Exclusion Diet (CDED) with partial enteral nutrition (PEN), have been proposed to induce remission in CD [119,120]. CDED consists of structured phases that remove components and additives harmful to the microbiota and intestinal barrier, while emphasizing high-quality proteins and foods that support microbial diversity. This diet is associated with reduced Pseudomonadota and increased Bacilliota [121]. A CDED study showed 76.7% remission by week 6, rising to 82.1% by week 12 [122]. In a trial involving children with mild-to-moderate CD, CDED led to remission, reduced inflammatory markers (C-reactive protein and ESR), fewer bowel movements, and lower fecal calprotectin levels. Participants also reduced their UPF intake and improved their adherence to a Mediterranean diet [123]. The diet’s maintenance phase is critical for developing and sustaining healthy eating habits, reinforcing the diet’s importance in gut health and microbiome [124].

## 7. Final Considerations

Excessive consumption of UPF can lead to various physiological alterations and impair gut microbiota function, contributing to the development of NCDs, including IBD. The impact of UPF on the gut microbiome and its association with the development and progression of IBD is a growing area of research. Highly consuming these foods promotes dysbiosis, increases intestinal permeability, and activates inflammatory pathways, which may exacerbate conditions such as CD and UC. Emerging evidence also supports the link between UPF consumption and increased IBD incidence, particularly CD. Conversely, diets rich in fresh, minimally processed foods promote gut health and may reduce inflammation, improving disease control in individuals with IBD. It is worth noting that few studies have evaluated the effect of UPF consumption on microbiota and the risk of IBD. Most studies have focused specifically on food additives, an area of knowledge essential for informing public policy and guiding regulatory measures. Modulating the gut microbiota through healthy dietary patterns represents a promising approach for preventing and managing IBD in at-risk populations. However, further research is necessary to clarify the underlying mechanisms and determine the most effective dietary interventions. Moreover, nutrition-based strategies aimed at modulating microbiota are expected to play an increasingly vital role in combating chronic diseases, enhancing patient quality of life, and reducing the broader economic and societal burdens associated with these conditions. The summary of the main articles of this review is shown in Table 4.

## 8. Study Limitations

Most studies presented herein were conducted on animals and in vitro models. Therefore, establishing a direct link between IBD pathophysiology and UPF requires clinical research designs, such as large-scale cohort studies. In particular, the association between azo dyes and intestinal inflammation demands more robust evidence, including data on the concentrations used in experimental models and how these correspond to the actual dietary exposure in the population. The pathophysiology of IBD is inherently multifaceted and cannot be attributed to a single factor; thus, its potential association with UPF—and specifically with azo dyes—is likely influenced by multiple, yet-to-be-elucidated determinants.

## Figures and Tables

**Table 1:** Summary of studies on food additives and the risk of gut disease.

Study (Year)	Model	Additive(s)	Key Findings Related to Gut Microbiota/Health
Suez et al., 2014 [36]	Germ-free mice	Non-caloric artificial sweeteners (NASs)	Excessive consumption may promote glucose intolerance, dysbiosis, and metabolic alteration.
Laudisi, 2018 [37]	Mice	Maltodextrin	Decreased Muc-2 results in greater adhesion of pathogenic bacteria.
Pinget et al., 2019 [38]	Mice	Titanium dioxide (TiO_2_)	TiO_2_ may impair intestinal homeostasis, increase inflammatory cytokine expression, and decrease crypt length.
He et al., 2021 [35]	Mice	Colorants Red 40 and Yellow 6	Can intensify intestinal inflammation and induce colitis.
Santos, 2023 [39]	Mice	Xanthan gum	Continuous consumption increases proinflammatory cytokines (TNF-α, IL-6, and IL-10) and alters intestinal barrier integrity.
Chassaing et al., 2022 [40]	Humans, 16 adults	Carboxymethylcellulose (CMC)	Alteration in gut microbiota composition and reduction of metabolites like SCFAs.
Araújo, 2017 [41]	Humans, ages 18–60 years	Carboxymethylcellulose (CMC)	Increased bacterial proliferation and infiltration, with an increase in *Roseburia* spp. and *Lachnospiraceae bacterium*.
Sieg et al., 2024 [42]	In vitro	Iron oxide food colorants (E 172)	E 172 showed strong interaction with intestinal cells, though no toxic effects were observed.
Han et al., 2025 [43]	In vitro	Carrageenan	Degraded carrageenan generates proinflammatory cytokines, such as IL-1β and TNF-α, which are related to the development of IBD.

**Table 2:** Description of the main altered phyla in patients with inflammatory bowel disease.

Phylum	Description	Reference
Bacteroidota and Bacillotas	Comprise 90% of the gut microbiota and are often reduced, potentially impairing the inflammatory response and short-chain fatty acid production.	Giambra et al. [61]; Santana et al. [64]
Proteobacteria	Typically increased, including opportunistic pathogens, such as Enterobacteriaceae and Burkholderiaceae, that can exacerbate inflammation.	Alam et al. [62]
Actinobacteria	In patients with Crohn’s disease, they are increased, which influences dysbiosis and intestinal inflammation.	Takahashi et al. [63]

**Table 3:** Altered bacterial profiles in Inflammatory Bowel Disease contribute to inflammation and exacerbation [64,65,66,67].

Increased in IBD		Decrease in IBD	
Phylum	Species	Phylum	Species
Proteobacteria [66]	*E. coli* *Campylobacter* spp. *H. parainfluenzae* *E. corrodens*	Verrucomicrobia [66]	*A. muciniphila*
Bacteroidota [64]	*B. fragilis*	Bacillota [65,67]	*F. prausnitzii* *R. albus* *Eubacterium* spp.
Bacillota [66]	*R. torques* *Ruminococcus* spp. *C. hathewayi* *C. bolteae* *R. gnavus*		

**Table 4:** The summary of the main articles of this review [7,16,17,19,21].

Study (Year)	Model	Objectives	Main Findings
Vanuytsel, 2021 [21]	Review	To review the role of the intestinal epithelial barrier in the pathophysiology of IBD.	Importance of epithelial integrity in preventing bacterial translocation and modulating the inflammatory response.
Babaei et al., 2022 [7]	Review	Evaluate the relationship between the intestinal microbiota and IBD.	Reduction of beneficial species and increase in pathogens in patients with IBD.
Whelean et al., 2024 [17]	Review	Explore mechanisms by which diet influences the intestinal microbiota and immunity.	Diets rich in sugars, saturated fat, and food additives promote dysbiosis and inflammation.
Cox et al., 2021 [16]	Review	Review the impact of additives and ultra-processed foods on the gut microbiota.	Emulsifiers, artificial sweeteners, and other additives can compromise the intestinal barrier and promote inflammation.
Zinöcker, 2018 [19]	Review	Integrate evidence on Western diet, microbiota, and IBD risk.	Reducing ultra-processed foods and increasing natural/minimally processed foods can contribute to the prevention and management of IBD.

## Abbreviations

The following abbreviations are used in this manuscript:

CMC	Carboxymethylcellulose
CD	Crohn’s disease
CDED	Crohn’s Disease Exclusion Diet
IBD	Inflammatory bowel disease
IL-1	Interleukin-1
IL-6	Interleukin-6
IL-10	Interleukin-10
IL-18	Interleukin-18
LPS	Lipopolysaccharide
MASLD	Metabolic-dysfunction-associated steatohepatitis
NAS	Non-caloric artificial sweetener
NCDs	Non-communicable chronic diseases
NF-kB	Nuclear factor kappa B
SCFAs	Short-chain fatty acids
SFA	Saturated fatty acid
TIO_2_	Titanium dioxide
TLR	Toll-like receptor
TNF-α	Tumor necrosis factor
UC	Ulcerative colitis
UPF	Ultra-processed food
